# Negative regulation of CD44st by miR-138-5p affects the invasive ability of breast cancer cells and patient prognosis after breast cancer surgery

**DOI:** 10.1186/s12885-023-10738-0

**Published:** 2023-03-24

**Authors:** Fang Xin Jian, Peng Xiao Bao, Wang Fu Li, Yan Hai Cui, Hang Guan Hong

**Affiliations:** 1 Department of Oncology, Gaochun Hospital Affiliated to Jiangsu University, Nanjing, China; 2 Department of Oncology, Lianyungang Hospital Affiliated to Jiangsu University, No.41, Hailian East Road, Lianyungang, 222000 Jiangsu China; 3 Bengbu Medical College, Bengbu, China

**Keywords:** miR-138-5p, CD44, Breast cancer, Invasion, Prognosis

## Abstract

**Objective:**

To investigate how the negative regulation of CD44st by miR-138-5p affects the invasive ability of breast cancer cell lines and prognosis in postoperative breast cancer patients.

**Methods:**

RT-PCR, qRT-PCR, and western blot assays were used to detect the expression of CD44s, CD44v6, and CD44st at both mRNA and protein levels. The expression of miR-138-5p in breast cancer cell lines was also evaluated. The binding ability of miR-138-5p to CD44st was determined via a dual-luciferase assay. The CD44 protein expression in breast cancer tissues was detected using immunohistochemistry. A Transwell assay was used to detect the invasive ability of tumor cells. The correlation between CD44st and miR-138-5p mRNA expression in breast cancer tissues was evaluated using qRT-PCR, and the relationship between clinicopathological features was statistically analyzed.

**Results:**

CD44s and CD44v6 were highly expressed in MDAMB-231 cell line, while CD44st was highly expressed in MCF-7/Adr and Skbr-3 cells. None of the CD44 isoforms were expressed in MCF-7 cells. The miR-138-5p was highly expressed in MCF-7 cells, but not in MCF-7/Adr, Skbr-3, and MDAMB-231 cells. The dual-luciferase assay suggested that miR-138-5p could bind to wild-type CD44st 3'-UTR, miR-138-5p overexpression significantly inhibited the expression level of CD44 protein in MCF-7/Adr cells, and miR-138-5p + CD44st (3'-UTR)-treated MCF-7/Adr and Skbr-3 cells were significantly less invasive than those in the control group (*P* < 0.05). RT-PCR results for 80 postoperative breast cancer patients showed that the mRNA expression rate for CD44st was higher in cancer tissues than in paracancerous tissues, and the expression rate of miR-138-5p was higher in paracancerous tissues than in cancerous tissues (*P* < 0.01). In cancer tissues, CD44st was negatively correlated with miR-138-5p expression, with correlation coefficient r = -0.76 (Pearson’s correlation), coefficient of determination R2 = 0.573, F = 106.89, and *P* < 0.001. The median overall survival value for patients in the low miR-138-5p expression group was 40.39 months [95% confidence interval (CI): 35.59–45.18 months] and 56.30 months (95% CI: 54.38–58.21 months) for patients in the high-expression group, with a log rank (Mantel-Cox) of 13.120, one degree of freedom, and *P* < 0.001.

**Conclusion:**

In breast cancer cell lines, miR-138-5p negatively regulated expression of CD44st and affected the invasive ability of tumor cells and patient prognosis after breast cancer surgery.

**Supplementary Information:**

The online version contains supplementary material available at 10.1186/s12885-023-10738-0.

MicroRNAs (miRNAs) are a class of small endogenous noncoding RNAs that regulate the expression of protein-coding genes at the level of transcription and exert biological functions by degrading or inhibiting mRNA expression [1, 2]. Studies have reported that miRNAs are widely involved in the biological behavior of cells and disease development, and their abnormal expression is closely related to tumor progression [3, 4]. These miRNAs can be used in the development of anti-cancer drugs and cancer biomarkers [5]. About 30% of the human genome can be regulated by miRNAs and most of them are located in the genomic regions or fragile gene loci associated with tumors [6, 7].

CD44 is a transmembrane glycoprotein that is a marker of tumor stem cells and is directly involved in tumor development [8]. It is expressed in connective tissue and cancer cells and is associated with tumor cell proliferation, differentiation, migration, angiogenesis, and chemoresistance [9–11]. Although abundant progress has been made regarding the structure and the expression of some CD44 isoforms, including CD44s and CD44v6, the abnormal expression of CD44 isoforms with clinicopathological impacts remains controversial. Therefore, it is important to explore the relationship among the expression of CD44 isoforms and the progression and prognosis in various types of cancers [8].

There are dozens of CD44 isoforms, which were shown in our previous study Fig. 1 [12]. In this study,we has identified CD44s, which is a new short-tailed form of CD44, and CD44st with mRNA ( National Center of Biotechnology Information NCBI Gene Bank FJ216964) containing bases 1–205 of exons 1–4, 16–17, and 18. CD44st was expressed in the breast cancer multidrug-resistant cell line MCF-7/Adr and not in the parental drug-sensitive cell line MCF-7. Further studies have confirmed that expression of CD44st was closely associated with multidrug resistance, invasion, breast cancer cell metastasis, and patient prognosis after breast cancer surgery [13, 14].

**Fig. 1:**
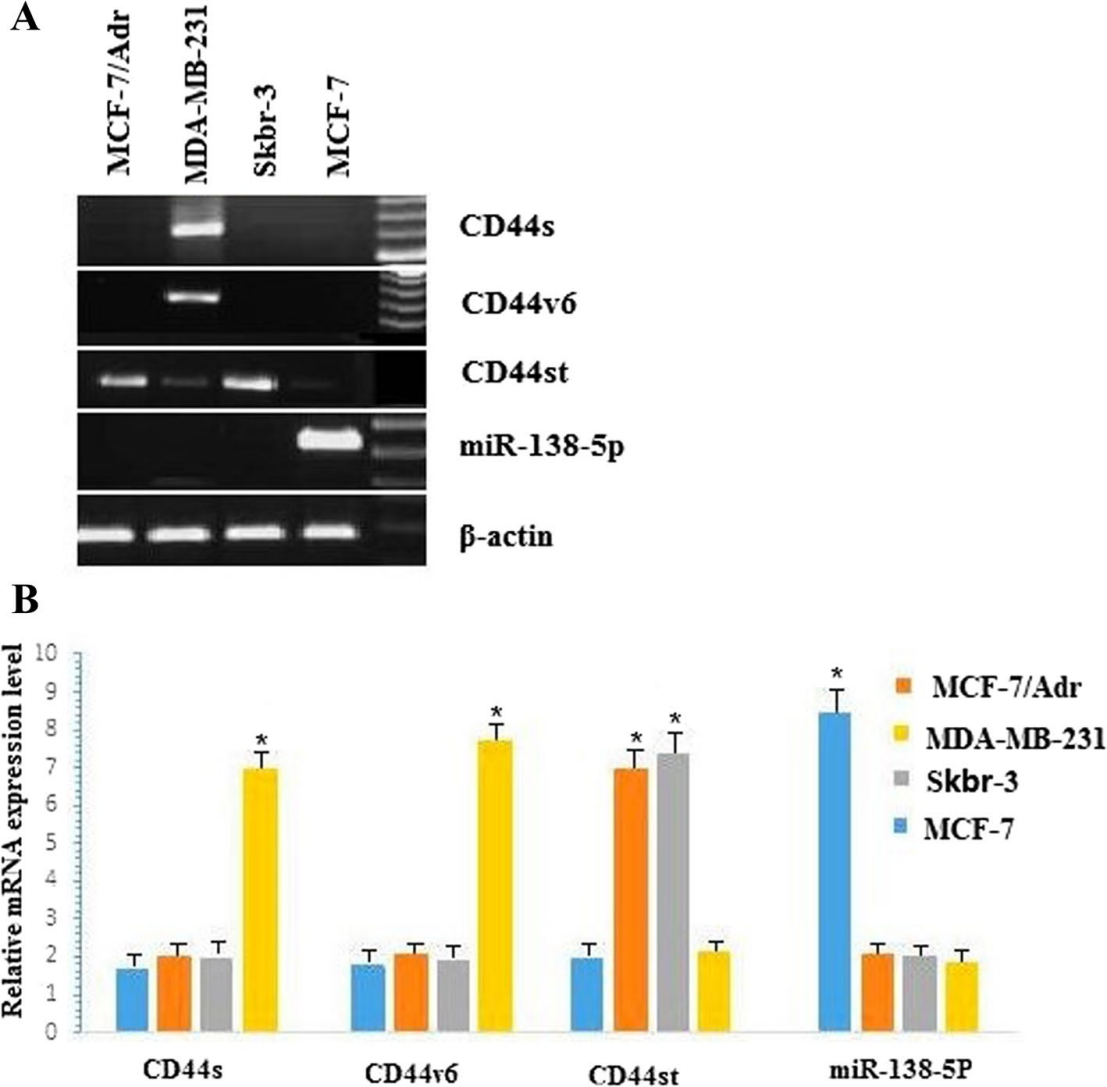
**A-B** Expression of CD44st, CD44s, CD44v6 mRNA and miR-138-5p in breast cancer cell lines. **A** Semi-quantitative PCR agarose electrophoresis plots of different cell lines **B **Quantitative PCR results of different cell lines

Recent studies have revealed that CD44 molecules played a key role in PD-L1 expression in breast and lung cancer cells. The intracytoplasmic region (ICD) of CD44 cleavage can regulate PD-L1 expression by binding to the coherence site of the PD-L1 gene’s promoter region.Therefore, CD44 is a potential target for inhibiting PD-L1 function in triple-negative breast cancer [15]. CD44 ICD can also interact with CREB to bind to the gene promoter region of PFKFB4, a key enzyme of glycolysis, to regulate the transcription and expression of PFKFB4, thereby regulating breast cancer cell stemness and affecting breast cancer patient prognosis [16]. In glioma cells, lactate-stimulated malignant glioma exosomes enhance the migratory and angiogenic effects of tumor cells through CD44, and there is a positive correlation between the malignant behavior of gliomas and their exosomal CD44 levels [16]. Meanwhile, CD44 is also involved in immune suppressor and promote glioma progression in glioma microenvironment [17].

Bioinformatics analysis showed that miR-138-5p directly targets CD44 by binding to the 3'-Untranslated Regions (3'-UTR) of CD44 mRNA according to the online database starBase (http://starbase.sysu.edu.cn/starbase2/index.php) and TargetScan software (http://www.targetscan.org/mamm_31/). Therefore, we hypothesized that miR-138-5p can target the negative regulation of CD44st expression, thereby inhibiting the proliferation and invasive ability of breast cancer cells. To test this hypothesis, molecular biology techniques, such as fluorescent quantitative PCR (Rq-PCR), western blot, and cell transfection, were utilized to investigate whether CD44st is a downstream target gene of miR-138-5p in breast cancer at the cellular, tissue, and clinical levels, the mechanism of the miR-138-5p’s effect on the invasive ability of breast cancer cells, and its influence on postoperative breast cancer patient prognosis.

## Materials and methods

### Experimental materials

1. Cell lines: MCF-7 (luminal A breast cancer cell line), MCF-7/Adr (parental adriamycin-resistant cell line of MCF-7), MDAMB-231 (triple-negative breast cancer cell line), and Skbr-3 (HER-2-overexpressing breast cell line) cell lines were purchased from the Shanghai Cell Bank of the Chinese Academy of Sciences. HEK293T cells were purchased from Shang Hai SXBIO Biotech Co, Ltd(Jinlang Road, Langxia Town, Jinshan District, Shanghai,China).
2. Patient inclusion criteria: Eighty post-radical breast cancer paraffin tissue specimens (Lianyungang Second People’s Hospital, Lianyungang Cancer Hospital) were collected between January 2015 and July 2016, including 67 cases of invasive ductal carcinoma and 13 cases of invasive lobular carcinoma. The corresponding paracancerous tissues 5 cm away from the tumor tissues were used as the negative controls.
3. Main reagents: Reverse transcription polymerase chain reaction (RT-PCR) kits, TRIzol, opti-MEM-I reduced serum medium (OPTI-MEM), Lipofectamine 3000 transfection kit, restriction endonuclease EcoRI, KpnI,paraffin-embedded tissue RNA extraction kit, and reverse transcription cDNA synthesis kit were purchased from NEB Corporation (USA). T4 ligase, high-fidelity Taq polymerase, 1640 medium, pMD20-T vector, Taq DNA polymerase (RR001Q), and fluorescent quantitative PCR kit (638,315) were purchased from TaKaRa Biologicals Ltd. MIR-138-5P mimic (5ʹ-AGCUGGUGUUGUUGAAUCAGGCCG-3ʹ) with the corresponding negative control (NC-miR, 5ʹ-GCGGUCGUGCAGUGCGUGAUAUA-3ʹ) was synthesized by RiboBio Inc. CD44 (AF6127), CD44s (BBA10), and CD44v6 (BBA13) antibodies were purchased from R&D systems (USA). Transwell assays were purchased from Corning, and Matrigel was purchased from BD. Paraffin-embedded tissue RNA extraction kit was purchased from Qiagen (73,504). Eukaryotic expression vector pcDNA3.1 was kindly provided by Prof. Wenrong Xu from Jiangsu University School of Medicine. The CD44st eukaryotic expression vector was constructed as described in a previous study [13]. The real-time quantitative PCR instrument was purchased from ABI (USA).

### Experimental methods

1. **CD44st plasmid construction and its expression in MCF-7/Adr cells was performed according to previous studies **[12]
2. **Identification of CD44s, CD44v6, CD44st, and miR-138-5p expression**
  MCF-7 (luminal A breast cancer cell line), MCF-7/Adr (parental adriamycin-resistant cell line of MCF-7), MDAMB-231 (triple-negative breast cancer cell line), and Skbr-3 (HER-2-overexpressing breast cell line) cell lines were used. Based on the sequences for different exons involved in shearing, specific primer sequences for CD44s, CD44st, and CD44v6 were designed, and the expression levels of CD44s, CD44st, and CD44v6 mRNA and miR-138-5p were investigated using RT-PCR and RT-qPCR. CD44s and CD44v6 protein expression was detected using western blotting. In our previous study, the CD44st-specific monoclonal antibody was not fully developed by Abimat Biomedical (Shanghai) Co, Ltd. due to the high homology level of this mRNA sequence. Therefore, CD44 protein expression was tested to verify the protein encoded by CD44st mRNA.
3. **Dual-luciferase reporter analysis**
  To clarify whether CD44st is regulated by miR-138-5p, PCR was used to amplify and embed a p-miR-reporter (Ambion, USA) to construct a CD44st-wild type (CD44st-WT 3ʹ-UTR) luciferase vector containing a WT human CD44st gene 3ʹ-UTR, assuming miR-138-5p binding with a DNA sequence. A mutant 3ʹ-UTR was similarly embedded in the p-miR-reporter, and a luciferase CD44st-mutant (CD44st-Mut 3ʹ-UTR) vector was constructed. HEK293T cells were transfected with mimics negative control (NC) or miR-138-5p mimics and then co-transfected with CD44st-WT 3ʹ-UTR or CD44st-Mut 3ʹ-UTR for 48 h. Relative fluorescence intensity was measured using a dual-luciferase reporter assay (Promega, USA) according to the operating instructions.
4. **Transwell assay for cell invasiveness**
  CD44st 3'-UTR and CD44st (no 3'-UTR) expression vectors were co-transfected with miR-138-5p mimics into logarithmic growth phase MCF-7/Adr and Skbr-3 cells, respectively. The two types of cells were divided into six groups: MCF-7/Adr group, MCF-7/Adr + miR-138-5p + CD44st (no 3'-UTR) group, MCF-7/Adr + miR-138-5p + CD44st (3'-UTR) group, Skbr-3 group, Skbr-3 + miR-138-5p + CD44st (no 3'UTR) group, and Skbr-3 + miR-138-5p + CD44st (3'-UTR) group. After transfection for 48 h, cells were collected from each experimental group, and 1.5 × 10^6^ cells were inoculated separately in culture flasks. Matrigel was diluted (1:1) using serum-free RPMI-1640 culture medium, added to the Transwell (150 µL/each), and incubated at 37℃ for 1 h to allow Matrigel to fully polymerize. Then, 1 mL of RPMI-1640 culture medium containing 10% neonatal bovine serum was added to the lower Transwell chamber. After 1 h, the cells in each experimental group were collected and prepared into a cell suspension of 10^5^ cells/mL. A total of 200 μL of cell suspension were inoculated into the upper Transwell chamber and cultured for 24 h. The Transwell was removed, the Matrigel was swabbed off the surface of the polycarbonate membrane, and the top and bottom of the polycarbonate membrane were gently rinsed with phosphate-buffered saline and dried.
  The excess Crystal Violet staining solution was removed after 10 min of 0.1% crystalline violet staining and the test samples were observed under the light microscope. Five fields of view at 200 × were randomly selected for each membrane, and the number of cells penetrating the membrane on the lower surface of the polycarbonate membrane was counted. The number of penetrating cells was used to indicate the invasive ability of tumor cells. The experiment was repeated three times.
5. **RNA extraction from paraffin-embedded tissue, semi-quantitative RT-PCR, and gene sequencing**
  Tumor RNA was extracted using paraffin-embedded tissue RNA extraction kit according to the manufacturer instructions. CD44st (GenBank NO, FJ 216,964) semi-quantitative RT-PCR primer sequences and miR-138-5p primer sequences are shown in Table 1. The cDNA synthesis conditions were: 42ºC for 60 min and 94ºC for 5 min for a total of 30 cycles. The PCR amplification conditions were: 94ºC for 60 s, 54ºC for 30 s, 72ºC for 60 s, and a final 72ºC for 10 min to end the reaction. The gene sequencing method was described in a previous study [13].
6. **The qPCR amplification of CD44st and miR-138-5p genes**
  The primer sequences for CD44st, β-actin, miR-138-5p, and U6 are shown in Table 1. The reaction system was as follows: 32.0 μL of double distilled H2O, 1.0 μL of dNTP, 5.0 μL of 10 × buffer, 4.0 μL of MgCl_2_ (25 mM), 2.0 µL each of upstream and downstream primers (10 pM), 2.0 μL of Taq DNA polymerase (5 U/μL), and 2.0 μL of cDNA template, for a total of 50 μL. Three replicate wells were provided for each sample. CD44st gene with β-actin and miR-138-5p with U6 were used as the internal references. Amplification conditions were as follows: 94 °C for 5 min; 94 °C for 5 s, and 60 °C for 40 s, for a total of 40 cycles. The qPCR reaction system for the experimental group was stable, reproducible, and without any non-specific amplification. Using the equation, where ΔCt value in tumor tissue = Ct target gene—Ctβ-actin/U6, yielded the relative expression of the target gene relative to the internal reference gene β-actin/U6. The relative RNA expression level was determined using the 2^−ΔΔCt^ method.
7. **Statistical analysis**

**Table 1:** PCR primer sequences

Genes	Primer	sequences	size (bp)
CD44st	Forward primer Reverse primer	5′-CCCTGCTACCAGACACTCA-3′ 5′- TGTTCACCAAATGCACCAT-3′	1023
CD44v6	Forward primer Reverse primer	5′-CAGGCAACTCCTAGTAGTAC-3′ 5′-CCAAGATGATCAGCCATTCTGG-3′	604
CD44s	Forward primer Reverse primer	5′-AAGACATCTACCCCAGCAAC-3′ 5′-CCAAGATGATCAGCCATTCTGG-3′	324
miR-138-5p	Forward primer Reverse primer	5'-GGGAGCTGGTGTGTGAAT-3' 5'-CCAGTGCCGCAGGGT AGGT-3';	106
β-actin	Forward primer Reverse primer	5’-CTCGCGCTACTCTCTCTTTC-3’ 5’-CATGTCTCGATCCCACTTAAC-3’	330
U6	Forward primer Reverse primer	5’-TGCATCTCCATCTTCTACCCAAT-3' 5’-CCGACTGTGAGTGCCACTGT-3'	134

Statistical analyses were performed using SPSS 16.0 software (SPSS Inc.; Chicago, IL, USA). The measurement data were expressed as average number ± standard deviation (x ± s). One-way ANOVA was used to analyze the differences between transfected and negative control groups and to compare the means between multiple groups. The Q test was used for two-way comparisons between groups, and chi-square test was used to detect the differences in mRNA expression rates of miR-138-5p and CD44st genes. The mean ΔCt values for miR-138-5p and CD44st mRNA expression were tested using a K-S test, where the *P*-values were 0.17 and 0.22, respectively, indicating that the data were normally distributed. Spearman’s correlation analysis was utilized to calculate the correlation between miR-138-5p and CD44st mRNA expression rates. Linear correlation analysis was used to detect the correlation between the mRNA expression for miR-138-5p and CD44st gene. One- and multi-way ANOVA were performed to compare miR-138-5p and CD44st in terms of patient's clinicopathological death.The Kaplan–Meier method was used for analysis difference of disease free survival (DFS) and Overall Surviva (OS). Death due to reasons unrelated to cancer and those loss of visit were treated as censored data. *P*-values of < 0.05 were considered statistically significant.All of the cell experiments were repeated in triplicate.

## Results

### Expression of CD44st, CD44s, CD44v6, and miR-138-5p in breast cancer cell lines

**Fig. 2:**
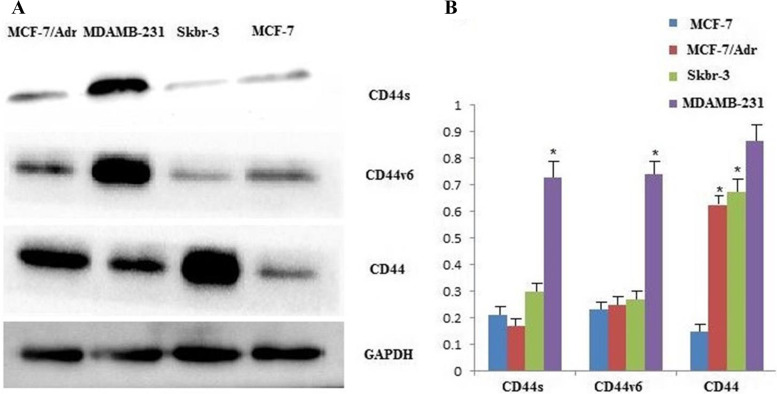
**A-B** Expression of CD44st, CD44s, CD44v6 proteins and miR-138-5p in breast cancer cell lines

It was found that miR-138-5p expression was significantly higher in MCF-7 cells than in other breast cancer cell lines (*P* < 0.05). In the MCF-7/Adr and Skbr-3 cell lines, CD44st mRNA expression was significantly higher than that in the MCF-7 and MDAMB-231 cell lines (*P* < 0.05). In the MDAMB-231 cell line, CD44s and CD44v6 mRNA expression was higher than that in the other two breast cancer cell lines (*P* < 0.05; Fig. 1A–B). Western blot analysis showed that CD44, CD44s, and CD44v6 proteins were highly expressed in the MDAMB-231 cell lines, and CD44 proteins were highly expressed in the MCF-7/Adr and Skbr-3 cell lines. This difference was statistically significant compared to the MCF-7 group (*P* < 0.05; Fig. 2A–B). These results suggested that CD44s and CD44v6 were highly expressed in the MDAMB-231 cell line, CD44st was highly expressed in the MCF-7/Adr and Skbr-3 cell lines, and none of the CD44 isoforms were expressed in the MCF-7 cell line. In addition, miR-138-5p was highly expressed in the MCF-7,but not in MCF-7/Adr, Skbr-3, and MDAMB-231 cell lines, suggesting a possible negative correlation between miR-138-5p and CD44 expression in breast cancer cells.

### Targeted regulation of CD44st by miR-138-5p

**Fig. 3:**
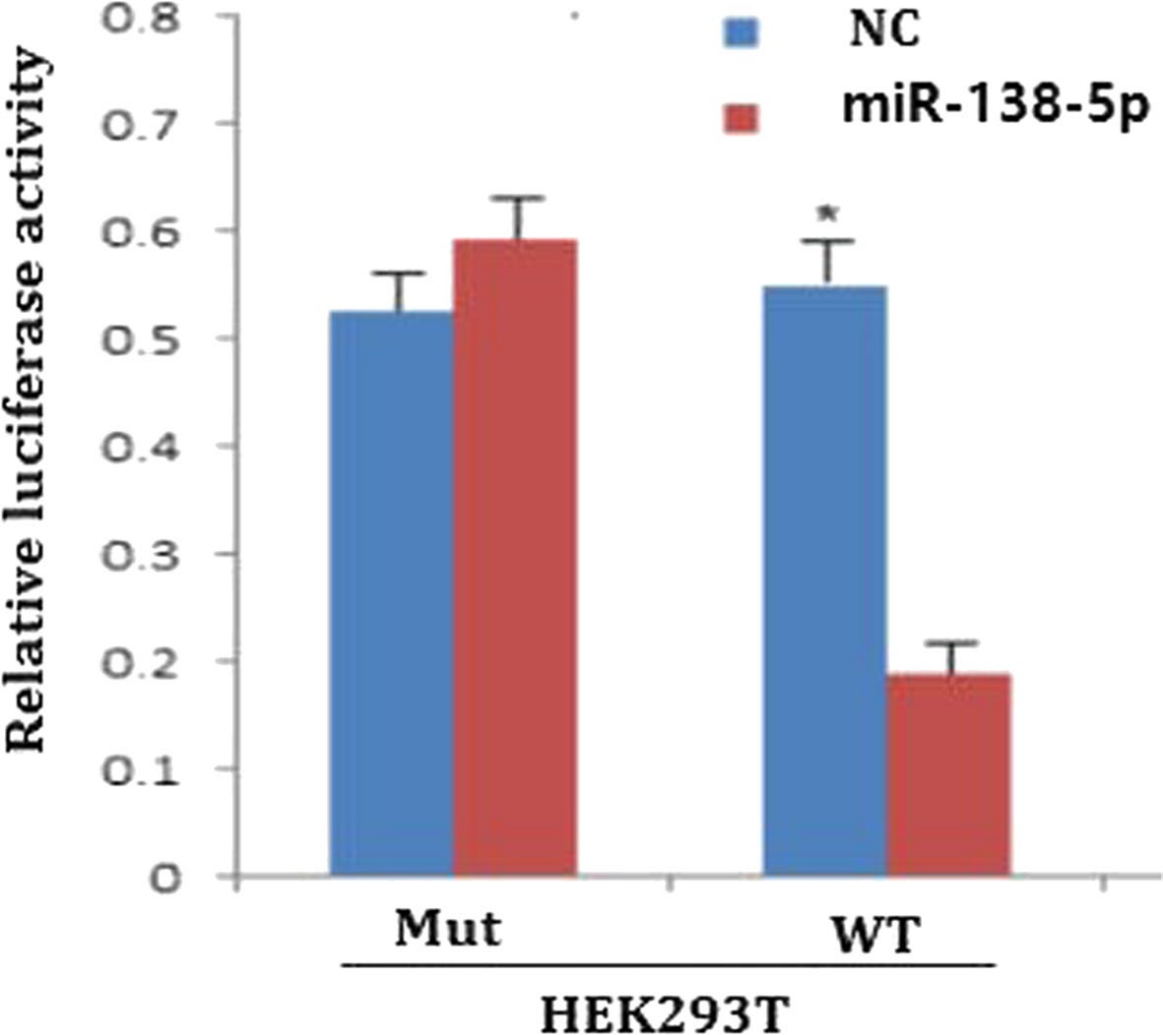
Targeted regulation of CD44st mRNA by miR-138-5p. WT, CD44st-WT 3ʹ-UTR Mut, CD44st-Mut 3ʹ-UTR

The results showed that miR-138-5p overexpression significantly reduced the luciferase activity of WT CD44st 3'-UTR reporter gene in the HEK293T cell line (*P* = 0.012), but had no significant effect on the luciferase activity of mutant CD44st 3'-UTR reporter gene. These results verified that miR-138-5p bound to WT CD44st 3'-UTR (Fig. 3).

### The miR-138-5p negatively regulates CD44 protein expression

**Fig. 4:**
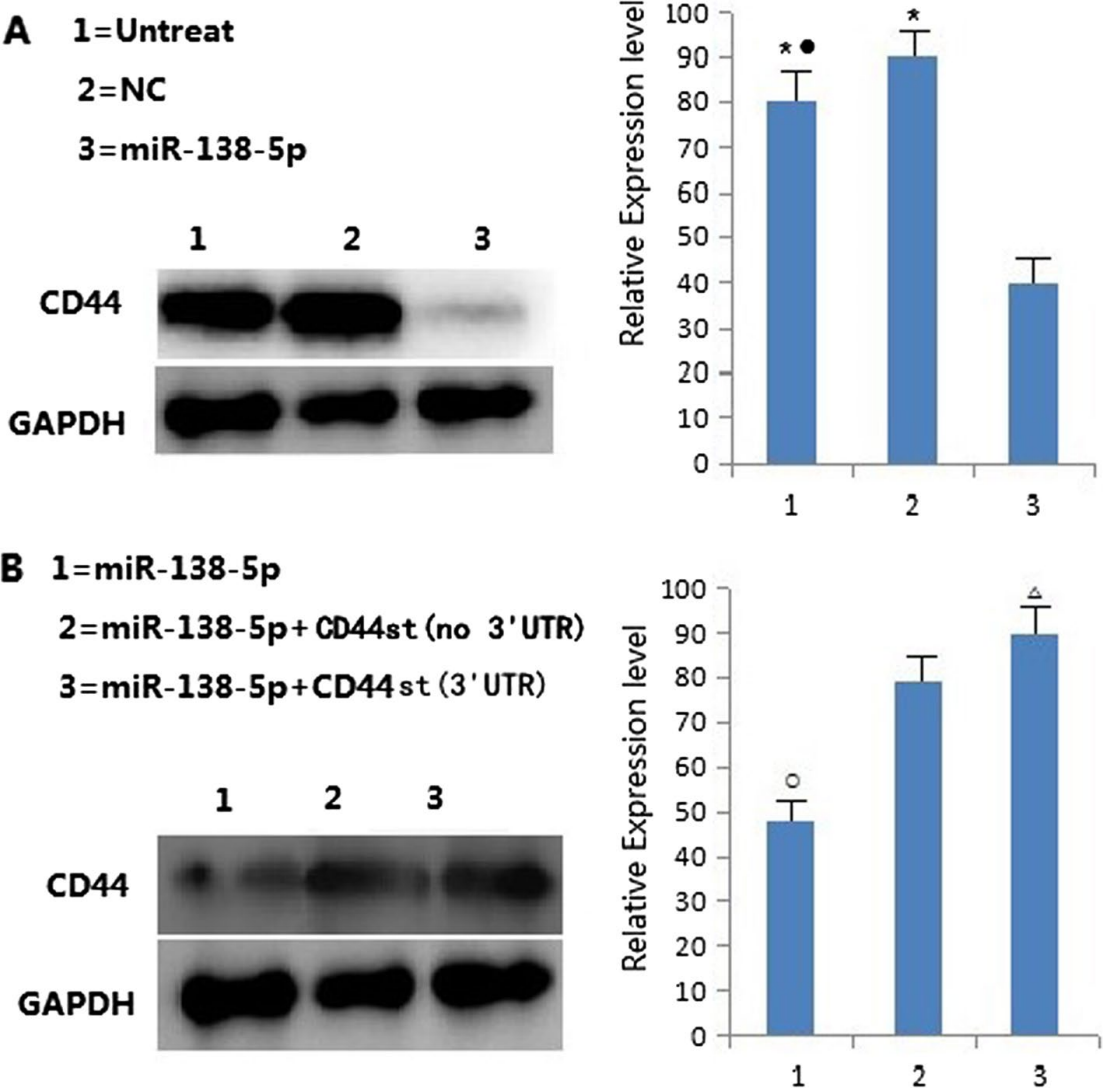
The miR-138-5p negatively regulates CD44 protein expression

In order to study the regulatory effect of miR-138-5p on CD44, MCF-7/Adr cell lines were transfected with mimics negative control (NC) and miR-138-5p mimics, respectively.The results showed that the expression of CD44 protein in MCF-7/Adr cells transfected with miR-138-5p mimics group was significantly lower than that in untreated and NC groups. CD44st 3'UTR and CD44st (no) expression vectors were co-transfected with miR-138-5p mimics into MCF-7/Adr cells, and the results showed that CD44st 3'-UTR and CD44st (no 3'-UTR) transfection attenuated the inhibitory effect of miR-138-5p on CD44 protein expression, while CD44st (no 3'-UTR) transfection had a more pronounced effect than CD44st 3'-UTR transfection. The present results further demonstrated that miR-138-5p overexpression significantly inhibited the expression level of CD44 protein in MCF-7/Adr cells (Fig. 4A–B).

### Effect of miR-138-5p on the invasion ability of MCF-7/Adr and Skbr-3 cells

**Fig. 5:**
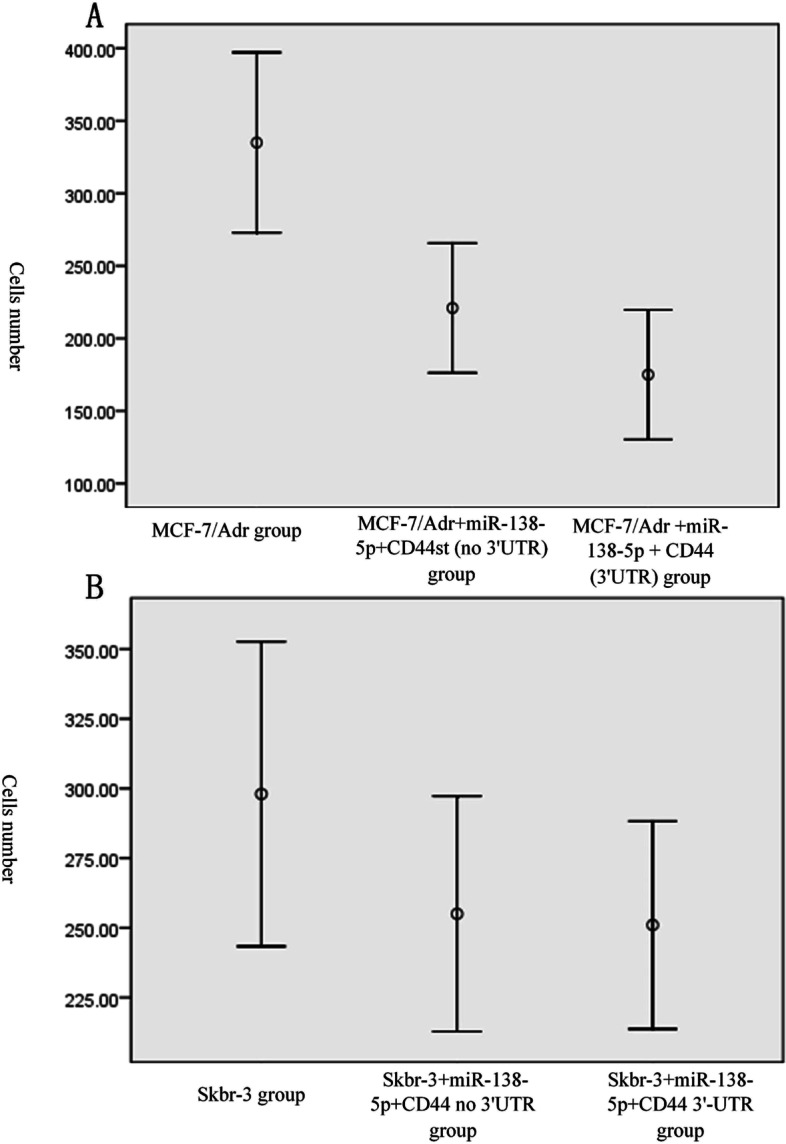
miR-138-5p overexpression inhibited the invasiveness of MCF-7/Adr and Skbr-3 cells as detected by transwell migration assay

The experimental results showed that the number of invasive cells was significantly higher in the MCF-7/Adr group (335 ± 25 cells/field of view) than in the MCF-7/Adr + miR-138-5p + CD44st (no 3'UTR) group (221 ± 18 cells/field of view) and MCF-7/Adr + miR-138-5p + CD44 (3'UTR) group (175 ± 18/field of view; Fig. 5 A1–3). The differences were statistically significant when comparing the three groups (*P* < 0.01). In the Skbr-3 group (298 ± 22 cells/field of view), the number of invasive cells was significantly higher than that in the Skbr-3 + miR-138-5p + CD44 no 3'UTR group (255 ± 17) and the Skbr-3 + miR-138-5p + CD44 3'-UTR group (241 ± 15; Fig. 5 B1–3). The difference between the Skbr-3 group and the remaining two groups was statistically significant (*P* = 0.036). The study results showed that miR-138-5p overexpression inhibited the invasiveness of MCF-7/Adr and Skbr-3 cells (Fig. 5).

### The miR-138-5p and CD44st gene expression in breast cancer and paracancerous tissues

**Fig. 6:**
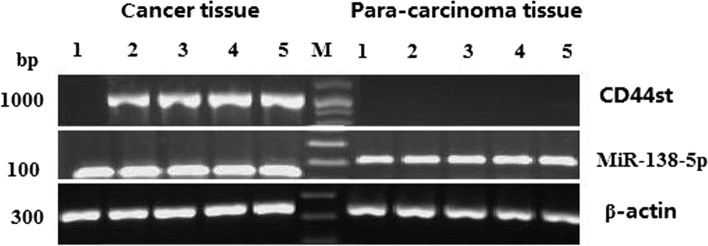
The miR-138-5p and CD44st mRNA expression in breast cancer and paracancerous tissues. Semi-quantitative PCR agarose electrophoresis plots of different tissues. (1–5: Breast cancer tissues and paracancerous tissues from five different patients, M: Marker)

The RT-PCR results for 80 patient specimens showed that miR-138-5p expression in breast cancer tissues was positive in 26 cases (32.5%) and negative in 54 cases (67.5%). CD44st expression was positive in 46 cases (57.5%) and negative in 34 cases (42.5%). In paraneoplastic tissues, miR-138-5p was positive in 55 cases (68.8%) and negative in 25 cases (31.2%), while CD44st was not expressed. The expression rate of CD44st mRNA was higher in cancer tissues than in paraneoplastic tissues, and the expression rate of miR-138-5p was higher in paraneoplastic tissues than in cancer tissues (*P* < 0.01; Table 2 and Fig. 6).

**Table 2:** The expression of CD44st and miR-138-5p in breast cancer tissues and paracancerous tissues, 80 cases (%)

	CD44st		miR-138-5p	
	+	-	+	-
paracancerous tissues	0 (0)	80 (100) ^*^	55 (68.8)	25 (31.2) ^*^
paracancerous tissues	46 (57.5)	34 (42.5) ^*^	26 (32.5)	25 (31.2) ^*^

P^*^ < 0.01

### Relationship between miR-138-5p and CD44st expression in breast cancer tissues

Subgroup analysis showed that miR-138-5p was highly expressed in five cases (10.9%, 5/46 cases) and had a low expression level in 41 cases (89.1%, 41/46 cases) in the CD44st mRNA-positive expression group. In the CD44st-negative expression group, miR-138-5p was highly expressed in 21 patients (61.8%, 21/34 cases) and had a low expression level in 13 patients (38.2%, 13/34 cases). The difference in miR-138-5p expression was statistically significant in the CD44st-positive expression group compared to the CD44st-negative expression group (*P* < 0.001). In addition, CD44st was negatively correlated with miR-138-5p expression (*r* = -0.537; Table 3).

**Table 3:** The expression of CD44st and miR-138-5p in breast cancer tissues,80 cases (%)

Groups	n	miR-138-5p +	miR-138-5p -
CD44 positive group	46	5 (10.9)	41 (89.1)
CD44 negative group	34	21 (61.8)	13 (38.2)

*P* < 0.001 Spearman Correlation *r* =—0.537

### CD44st gene sequencing

**Fig. 7:**
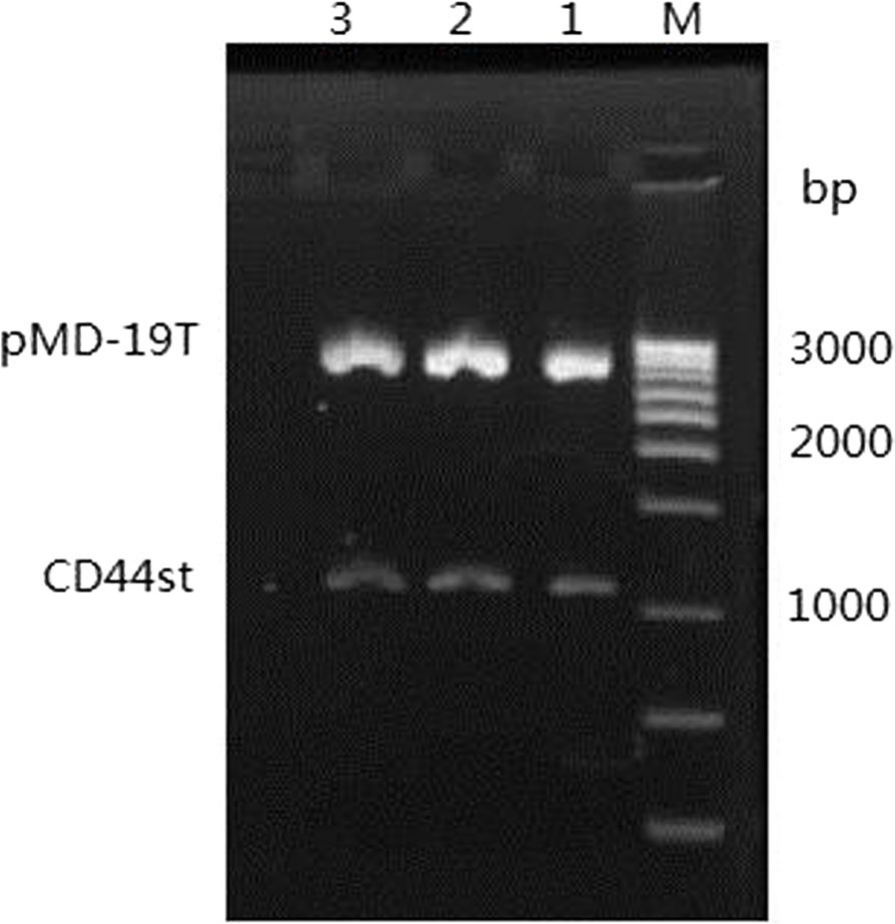
CD44st gene sequencing

The primer design software primer 5.0 was used to design the primers for human CD44st-1 (GeneBank NO. FJ 216,964) containing restriction endonuclease sites KpnI and EcoRIand β-actin gene fragment.The gene primer sequences for CD44st and β-actin are shown in Table 1. The PCR-amplified products were electrophoresed on 1.5% agarose gels and scanned using a gel imager, where the β-actin gene was used as an internal reference for quality control and standardization. The CD44st PCR amplification product was recovered and purified according to the gel recovery kit instructions. After T-vector cloning and transformation into *E. coli*, the plasmid was extracted and verified using KpnI and EcoRI double digestion. The positive clones were sent to Shanghai Bioengineering Technology Service Co., Ltd. for sequencing verification. The CD44st was confirmed to be consistent with the mRNA sequence for the found CD44 gene (Fig. 7).

### Correlation analysis of miR-138-5p and CD44st gene mRNA expression in breast cancer tissues

**Fig. 8:**
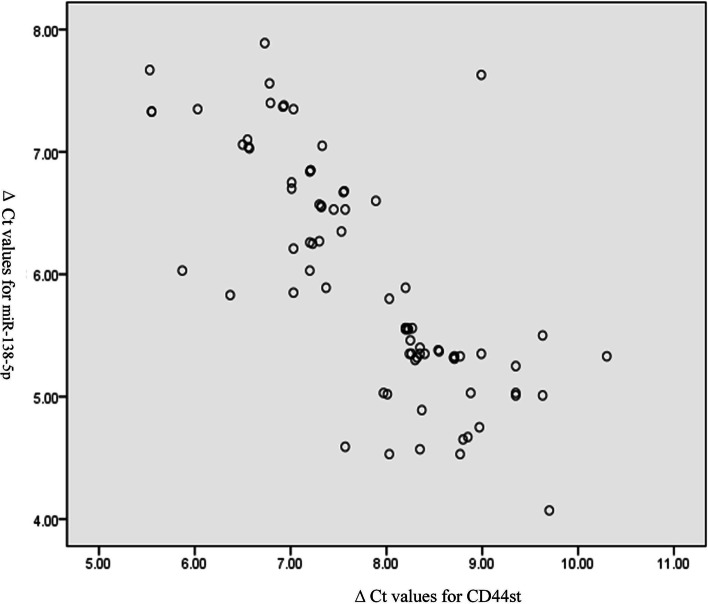
CD44st was negatively correlated with miR-138-5p gene mRNA expression

The median ΔCt values for CD44st and miR-138-5p gene mRNA in breast cancer tissues after labeling with the internal reference genes were 8.27 and 5.80, respectively. Correlation analysis showed that CD44st was negatively correlated with miR-138-5p gene mRNA expression, with correlation coefficient *r* = -0.76 (Pearson’s correlation), coefficient of determination R2 = 0.573, F = 106.89, and *P* < 0.001 (Fig. 8).

### Relationship between miR-138-5p and CD44st expression and clinicopathological characteristics

The miR-138-5p and CD44st mRNA expression was closely related to tumor TNM stage, lymph node metastasis, and molecular typing (*P* < 0.05). There was no significant relationship between miR-138-5p and CD44st mRNA expression and patient age, pathological type, and tumor size (*P* > 0.05). When the ΔCt values for CD44st and Her-2 mRNA expression were used as dependent variables, multifactorial Cox regression analysis further validated the above findings (miR-138-5p, R2 = 0.766; CD44st, R2 = 0.774; *P* < 0.05).

### Effect of miR-138-5p expression on disease free survival(DFS) and overallsurvival(OS)

**Fig. 9:**
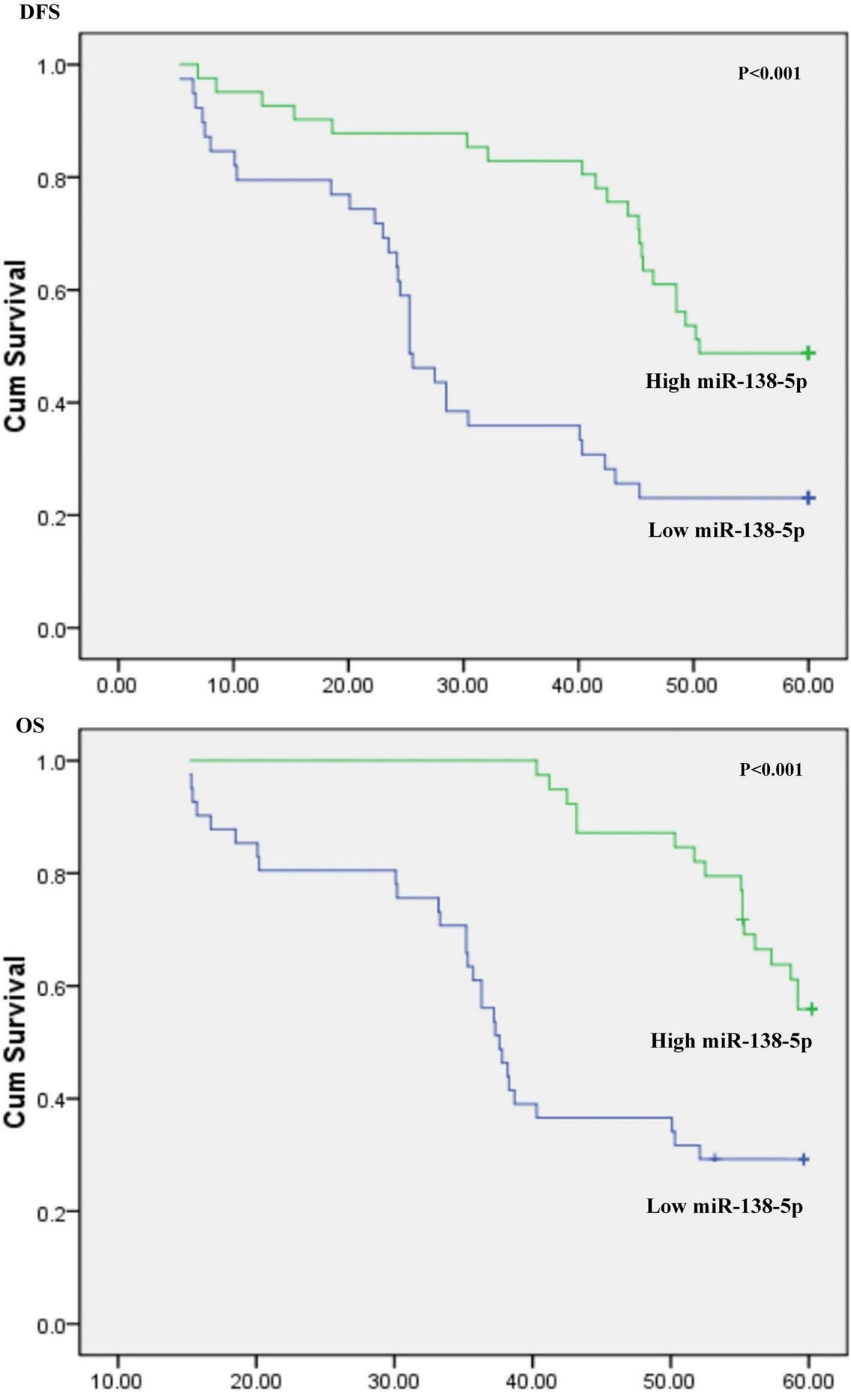
**A-B** Effect of miR-138-5p expression on disease free survival (DFS) and overallsurvival (OS)

ROC curves were used to determine the expression of miR-138-5p and CD44st mRNA in tumor tissues, and the corresponding maximum Youden index was the optimal cut-off value. The optimal cut-off ΔCt values for miR-138-5p and CD44st mRNA were 5.57 and 8.21, respectively. The median DFS was 31.67 months (95% CI: 25.86–37.47 months) for patients in the low miR-138-5p expression group and 47.99 months (95% CI: 43.19–52.80 months) for patients in the high miR-138-5p expression group, with log-rank (Mantel-Cox) of 12.18, one degree of freedom, and *P* < 0.001. The median OS was 40.39 months (95% CI: 35.59–45.18 months) for patients in the low miR-138-5p expression group and 56.30 months (95% CI: 54.38–58.21 months) for patients in the high-expression group, with log-rank (Mantel-Cox) of 13.120, one degree of freedom, and *P* < 0.001 (Fig. 9).

## Discussion

Previous studies have suggested that miR-138-5p was involved in the regulation of tumorigenesis, progression, and invasive ability in a variety of tumors. In Wei Zhang's study, miR-138-5p overexpression in gastric cancer cells inhibited the growth, proliferation, and migration and promoted apoptosis of gastric cancer cells by binding to the 3'-UTR of proto-oncogene DEK, while downregulation of miR-138-5p expression was closely associated with lymph node metastasis in gastric cancer patients [18]. CircRNA circGSE1 is highly expressed in cervical cancer tissues and is closely related to tumor differentiation, FIGUREO staging, infiltration depth, lymph node metastasis, parametrial infiltration, and patient prognosis. In addition, miR-138-5p can bind to the 3'-UTR of vimentin protein mRNA and inhibit its expression, thus inhibiting tumor cell invasion and metastasis, while circGSE1 can directly bind and adsorb miR-138-5p and enhance vimentin protein expression to reverse the above effects [19]. Long non-coding RNA HMMR-AS1, a competing endogenous RNA (ceRNA) for miR-138, is highly expressed in lung adenocarcinoma tissues, promotes tumor proliferation and invasion, and is closely associated with tumor size, TNM stage, and prognosis, while HMMR-AS1-miR-138-sirt6 plays an important role in the origin of lung cancer [20]. There is still a controversy about the role of miR-138-5p in tumor invasion and metastasis. Liang-Yan Chen has reported that the circ-ERBIN-mediated miR-125a-5p-5p/miR-138-5p/4EBP-1 axis activated the key mechanism of HIF-1α, which increased the proliferation and migration ability of intestinal cancer cells, suggesting that circ-ERBIN could be a potential target for Colorectal Cancer CRC therapy [21]. Trp53 is a direct target of miR-138-5p, and miR-138-5p overexpression can lead to enhanced metastatic ability of melanoma cells by inhibiting Tp53 expression [22].

The miR-138-5p has a low expression in breast cancer tissues and cells. Overexpression of miR-138-5p targets rhomboid domain-containing protein 1 and significantly inhibits the invasion, migration, and epithelial-mesenchymal transition (EMT) of breast cancer cells by upregulating E-cadherin expression and downregulating N-cadherin and vimentin [23]. In breast cancer cells, miR-138 acts as a novel regulator targeting histone methylation transferase to regulate tumor invasion and EMT and cycle-dependent kinase inhibitor-related protein to regulate proliferation and migration of breast cancer cells [24]. The miR-138-activated signal thus might serve as a novel independent prognostic marker [25].

Recent studies have confirmed CD44 + /CD24- phenotype in breast cancer patients was negatively correlated with postoperative DFS and OS [26]. It was found that there were significant differences between different subtypes of CD44 that affected the biological behavior of breast cancer cells, among which the subtypes associated with breast cancer invasion, metastasis, and patient prognosis mainly included CD44s, CD44st, CD44v6, and CD44v9. Studies have reported that CD44s were more closely related to HER-2 or basal cell subtypes in molecular typing of breast cancer [27] and were significantly associated with tumor aggressiveness and poor prognosis in breast cancer with axillary lymph node metastases [28]. And CD44v6 expression are potential predictors of metastases of the lymphatic node in invasive breast carcinoma of no special type [29]. In addition, CD44v6 is closely associated with histological grading, lymph node metastasis, and poor prognosis in breast cancer patients [30]. CD44v9 expression is associated with poor patient prognosis in triple-negative breast cancer [31].

The expression of the adhesion molecule CD44 can be influenced by many factors, including chemotherapeutic agents, cellular molecules, and intracellular signaling. MiRNAs are also involved in the expression and regulation of CD44. Studies have reported that miRNAs, such as miRNA-150 [32], miRNA-34a [33], miRNA-200c [34], miRNA-330-5p [35], and miRNA-143 [36], negatively regulated CD44 expression in nasopharyngeal, esophageal, ovarian, non-small cell lung, and breast cancers, inhibiting the invasive and metastatic ability of tumor cells and tumor stemness. In malignant glioma patients, whole transcriptome and miRNA expression profiling showed that miR-138 was negatively regulated and its expression level was negatively correlated with the expression level of CD44 molecules. Transient transfection of miR-138 inhibited the proliferation, cell cycle, and migration ability and decreased invasion ability of glioma cells. In glioma cells, miR-138 can inhibit CD44 expression by binding to the 3'-UTR of CD44 mRNA, causing activation of the cell cycle inhibitory factor P27 and nuclear translocation, as well as cell cycle arrest in the G0 or G1 phase, leading to downregulation of the proliferative capacity in glioma cells by inhibiting the CD44/AKT signaling pathway [37, 38].

However, some other miRNAs have also been found to positively regulate CD44 expression affecting the pathobiological features of tumors. MiRNA-492 positively regulates CD44 (CD44s and CD44v10) expression and increases the proliferation, cell adhesion, and invasive ability of hepatoblastoma cells. High expression of miR-492 is associated with high invasive characteristics of tumors and poor prognosis [39]. In ovarian cancer tissues and cells, miRNA-21 increases CD44v6 expression by activating the Wnt signaling pathway, which in turn promotes tumor cell proliferation, invasion, and migration ability [40].

The present study examined the expression of CD44s, CD44v6, and CD44st at both mRNA and protein levels and miR-138-5p at RNA level in breast cancer cell lines and postoperative breast cancer tissues. The binding ability of miR-138-5p to CD44st was verified using a dual-luciferase assay. The correlation between CD44st and miR-138-5p mRNA expression in breast cancer tissues and the effect of miR-138-5p expression on the prognosis of postoperative breast cancer patients were examined. It was found that CD44s and CD44v6 were highly expressed in MDAMB-231 cells, CD44st was highly expressed in MCF-7/Adr and Skbr-3 cells, and all isoforms of CD44 were not expressed or had a low expression level in MCF-7 cells. In MCF-7/Adr and Skbr3 cells, miR-138-5p could bind to the 3'-UTR of WT CD44 and inhibit the CD44 protein expression level in MCF-7/Adr cells. The miR-138-5p + CD44 (3'UTR) treatment resulted in a significantly lower invasion ability of MCF-7/Adr and Skbr-3 cells than that of control cells. The expression rate of CD44st mRNA was higher in cancer tissues than in paraneoplastic tissues, while the expression rate of miR-138-5p was higher in paraneoplastic tissues than in cancer tissues. CD44st in cancer tissues was negatively correlated with miR-138-5p expression, with a median OS of 40.39 months (95% CI: 35.59–45.18 months) for patients in the low miR-138-5p expression group and 56.30 months (95% CI: 54.38–58.21 months) for patients in the high miR-138-5p expression group. The expression level of miR-138-5p was positively correlated with patient survival time.

In conclusion, miR-138-5p regulated the expression of CD44st in breast cancer cells, affected patient prognosis after breast cancer surgery, and could be used as a biomarker to assess patient prognosis after breast cancer surgery.

## Supplementary Information

**Additional file 1: Figure 1A-B.** Expression of CD44st, CD44s, CD44v6 mRNA and miR-138-5p in breast cancer cell lines. A: Semi-quantitative PCR agarose electrophoresis plots of different cell lines B:Quantitative PCR results of different cell lines.

**Additional file 2: Figure 2A-B. **Expression of CD44st, CD44s, CD44v6 proteins and miR-138-5p in breast cancer cell lines.

**Additional file 3. **

**Additional file 4: Figure 4. **The miR-138-5p negatively regulates CD44 protein expression.

**Additional file 5: Figure 6.** The miR-138-5p and CD44st mRNA expression in breast cancer and paracancerous tissues.

**Additional file 6: Figure 7. **CD44st gene sequencing.

## Data Availability

The datasets generated and/or analysed during the current study are available in the Hospital Technology Office or the corresponding author on reasonable request
