# Supplementary material for: Negative regulation of CD44st by miR-138-5p affects the invasive ability of breast cancer cells and patient prognosis after breast cancer surgery
Source: BMC Cancer. 2023 Mar 24;23:269. doi: 10.1186/s12885-023-10738-0 (PMC10037889; doi:10.1186/s12885-023-10738-0)
Supplement: Supplementary file 2 — Additional file 2: Figure 2A-B. Expression of CD44st, CD44s, CD44v6 proteins and miR-138-5p in breast cancer cell lines. [file 12885_2023_10738_MOESM2_ESM.docx]

**Figure. 2A-B.** Expression of CD44st, CD44s, CD44v6 proteins and miR-138-5p in breast cancer cell lines.


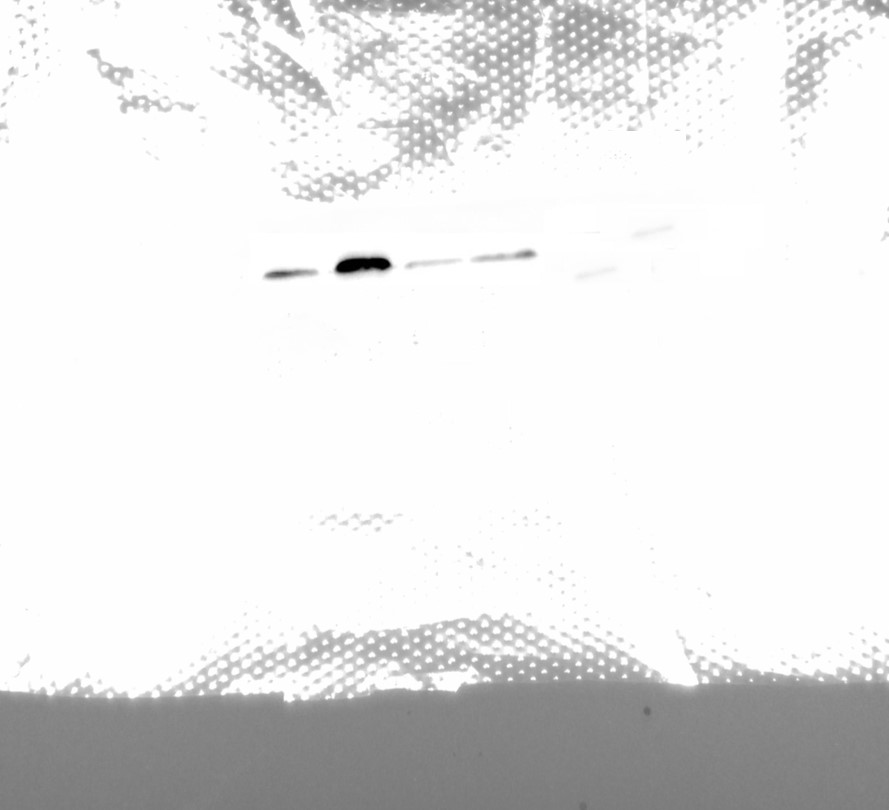
 CD44s


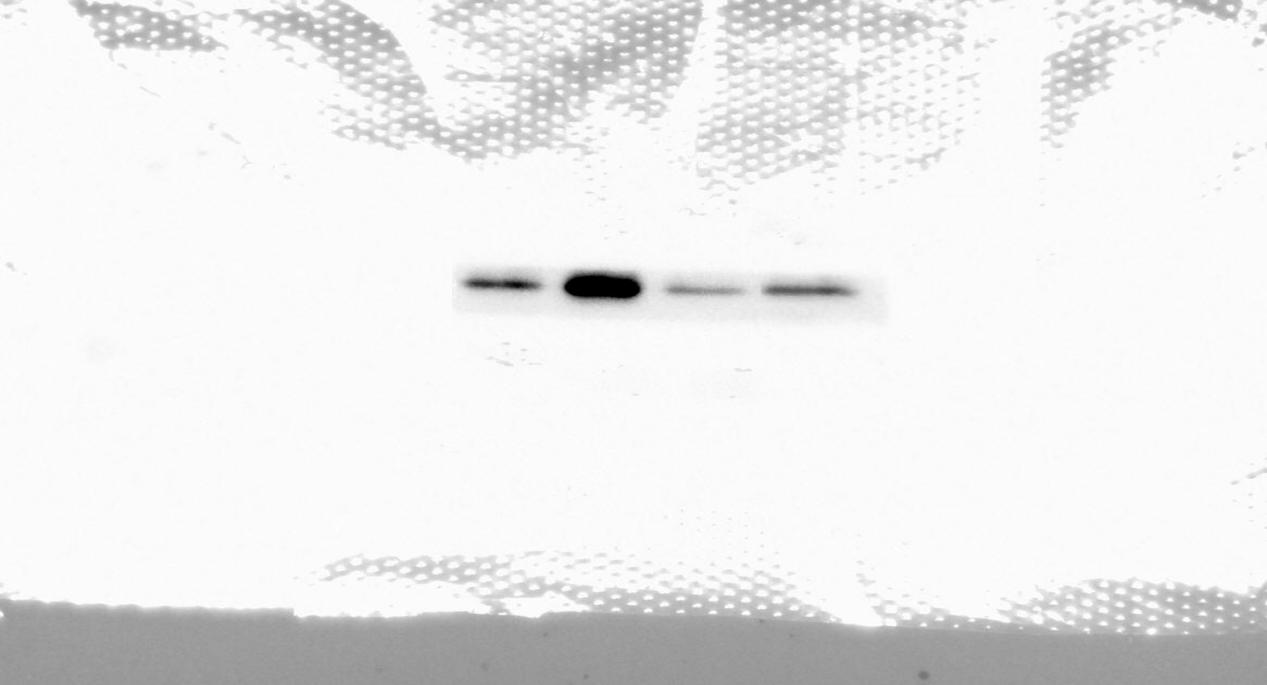


CD44v6


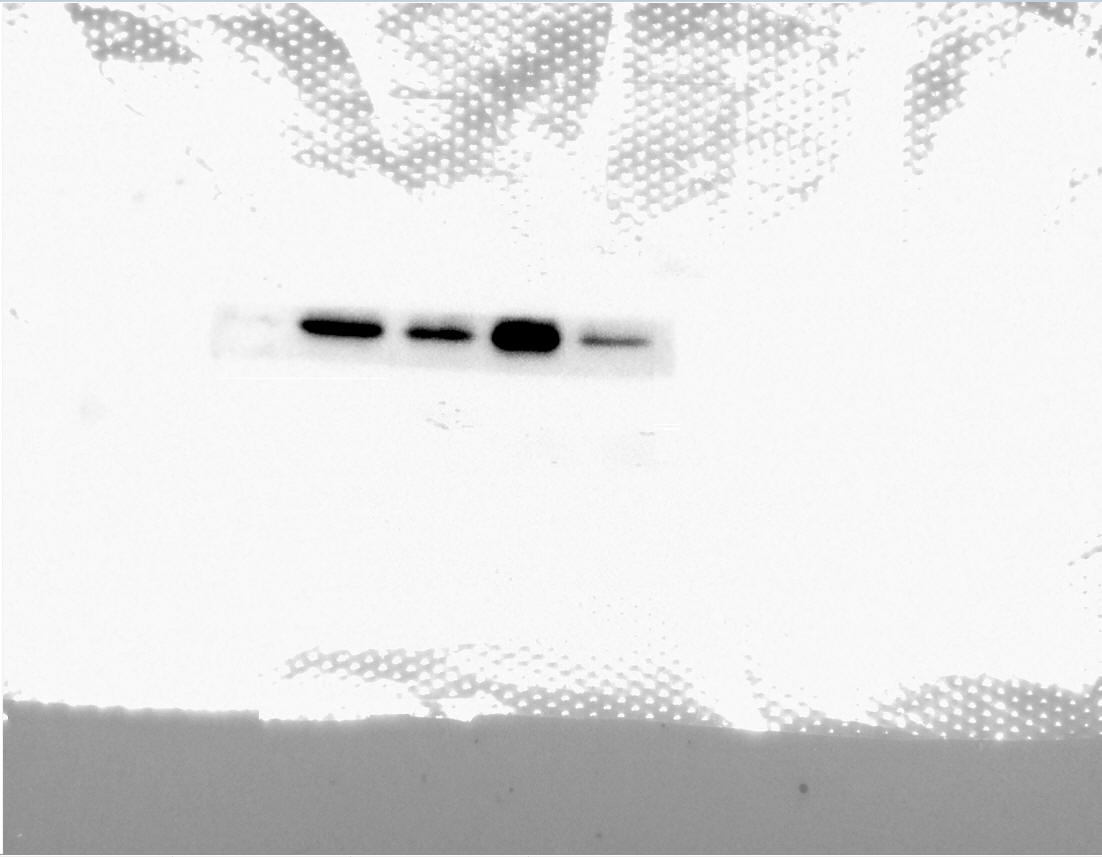


CD44


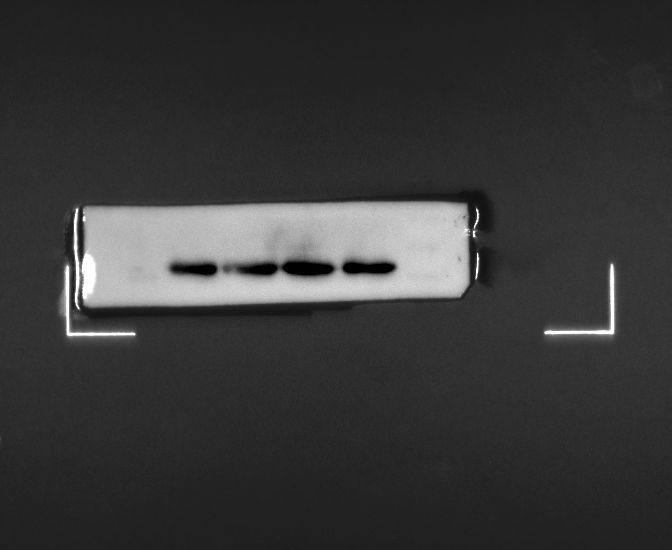


GAPDH
