# Supplementary material for: Negative regulation of CD44st by miR-138-5p affects the invasive ability of breast cancer cells and patient prognosis after breast cancer surgery
Source: BMC Cancer. 2023 Mar 24;23:269. doi: 10.1186/s12885-023-10738-0 (PMC10037889; doi:10.1186/s12885-023-10738-0)
Supplement: Supplementary file 3 — Additional file 3. [file 12885_2023_10738_MOESM3_ESM.pdf]

Block Type	96well
Chemistry	SYBR_GREEN
Experiment	D:\Applied Biosystems\StepOne Software v2.2.3\experiments\wangfuli\20220621.eds
Experiment	2022-06-21 19:25:02 PM CST
Instrument	steponeplus
Passive Re	ROX

Well	Sample Na	Target Nan	Task	Reporter	Quencher	Cт	Cт Mean	Cт SD
A1	U6							
A2								
A3								
A4								
A5	U6	MUT-NC	UNKNOWN	SYBR	None	18.59783173	18.68333626	0.074099
A6	U6	MUT-NC	UNKNOWN	SYBR	None	18.72339249	18.68333626	0.074099
A7	U6	MUT-NC	UNKNOWN	SYBR	None	18.72878838	18.68333626	0.074099
A8								
A9								
A10								
A11								
A12								
B1								
B2								
B3								
B4								
B5	U6	MUT-138-5	UNKNOWN	SYBR	None	19.65108681	19.69213295	0.036422
B6	U6	MUT-138-5	UNKNOWN	SYBR	None	19.70472527	19.69213295	0.036422
B7	U6	MUT-138-5	UNKNOWN	SYBR	None	19.72059059	19.69213295	0.036422
B8								
B9								
B10								
B11								
B12								
C1								
C2								
C3								
C4								
C5	U6	WT-NC	UNKNOWN	SYBR	None	18.84844589	18.913908	0.062696
C6	U6	WT-NC	UNKNOWN	SYBR	None	18.91986275	18.913908	0.062696
C7	U6	WT-NC	UNKNOWN	SYBR	None	18.97341347	18.913908	0.062696
C8								
C9								
C10								
C11								
C12								
D1								
D2								
D3								
D4								
D5	U6	WT-138-5F	UNKNOWN	SYBR	None	18.94997597	18.96536255	0.013351
D6	U6	WT-138-5F	UNKNOWN	SYBR	None	18.97388649	18.96536255	0.013351
D7	U6	WT-138-5F	UNKNOWN	SYBR	None	18.97222519	18.96536255	0.013351
D8								

D9								
D10								
D11								
D12								
E1								
E2								
E3								
E4								
E5	CD44st	MUT-NC	UNKNOWN	SYBR	None	23.55541992	23.71026802	0.194147
E6	CD44st	MUT-NC	UNKNOWN	SYBR	None	23.64730263	23.71026802	0.194147
E7	CD44st	MUT-NC	UNKNOWN	SYBR	None	23.92808342	23.71026802	0.194147
E8								
E9								
E10								
E11								
E12								
F1								
F2								
F3								
F4								
F5	CD44st	MUT-138-5	UNKNOWN	SYBR	None	22.93444633	22.11049843	0.170285
F6	CD44st	MUT-138-5	UNKNOWN	SYBR	None	22.1226902	22.11049843	0.170285
F7	CD44st	MUT-138-5	UNKNOWN	SYBR	None	22.27436066	22.11049843	0.170285
F8								
F9								
F10								
F11								
F12								
G1								
G2								
G3								
G4								
G5	CD44st	WT-NC	UNKNOWN	SYBR	None	23.11261368	23.3271389	0.193266
G6	CD44st	WT-NC	UNKNOWN	SYBR	None	23.48765755	23.3271389	0.193266
G7	CD44st	WT-NC	UNKNOWN	SYBR	None	23.38114166	23.3271389	0.193266
G8								
G9								
G10								
G11								
G12								
H1								
H2								
H3								
H4								
H5	CD44st	WT-138-5F	UNKNOWN	SYBR	None	30.16150284	30.41297531	0.656722
H6	CD44st	WT-138-5F	UNKNOWN	SYBR	None	30.14389038	30.41297531	0.656722
H7	CD44st	WT-138-5F	UNKNOWN	SYBR	None	30.93353081	30.41297531	0.656722
H8								
H9								
H10								
H11								
H12								

Quantity	Quantity M	Quantity SI	Automatic (	Ct Thresho	Automatic I	Baseline St	Baseline Er	Tm1
			TRUE	1.530902	TRUE	3	10	80.75836
			TRUE	1.530902	TRUE	3	10	80.75836
			TRUE	1.530902	TRUE	3	10	80.61037
			TRUE	1.335104	TRUE	3	12	80.60904
			TRUE	1.335104	TRUE	3	12	80.60904
			TRUE	1.335104	TRUE	3	12	80.46102
			TRUE	1.316051	TRUE	3	11	80.75836
			TRUE	1.316051	TRUE	3	11	80.75836
			TRUE	1.316051	TRUE	3	11	80.61037
			TRUE	1.253751	TRUE	3	11	80.60904
			TRUE	1.253751	TRUE	3	11	80.45972
			TRUE	1.253751	TRUE	3	11	80.46102

TRUE	1.530902	TRUE	3	22	84.64075
TRUE	1.530902	TRUE	3	22	84.49142
TRUE	1.530902	TRUE	3	23	84.3443

TRUE	1.335104	TRUE	3	23	84.3421
TRUE	1.335104	TRUE	3	23	84.49142
TRUE	1.335104	TRUE	3	23	84.3443

TRUE	1.316051	TRUE	3	23	84.3421
TRUE	1.316051	TRUE	3	24	84.19278
TRUE	1.316051	TRUE	3	23	84.19495

TRUE	1.253751	TRUE	3	25	84.04346
TRUE	1.253751	TRUE	3	24	84.19278
TRUE	1.253751	TRUE	3	24	84.19495

Tm2	Tm3	Comments	HIGHSD
			N
			N
			N
			N
			N
			N
			N
			N
			N
			N
			N
			N
			N
			N
			N
			N
			N
			N
			N
			N
			N
			N
			N
			N
			N
			N
			N
			N
			N
			N
			N
			N
			N
			N
			N
			N
			N
			N
			N
			N
			N
			N
			N
			N

N
N
N
N
N
N
N
N
N
N
N
N
N
N
N
N
N
N
N
N
N
N
N
N
N
N
N
N
N
N
N
N
N
N
N
N
N
N
N
N
N
N
N
N
Y
Y
Y
N
N
N
N
N
