# Supplementary figures and images for: Negative regulation of CD44st by miR-138-5p affects the invasive ability of breast cancer cells and patient prognosis after breast cancer surgery
Source: BMC Cancer. 2023 Mar 24;23:269. doi: 10.1186/s12885-023-10738-0 (PMC10037889; doi:10.1186/s12885-023-10738-0)

**Figure.4.** The miR-138-5p negatively regulates CD44 protein expression.


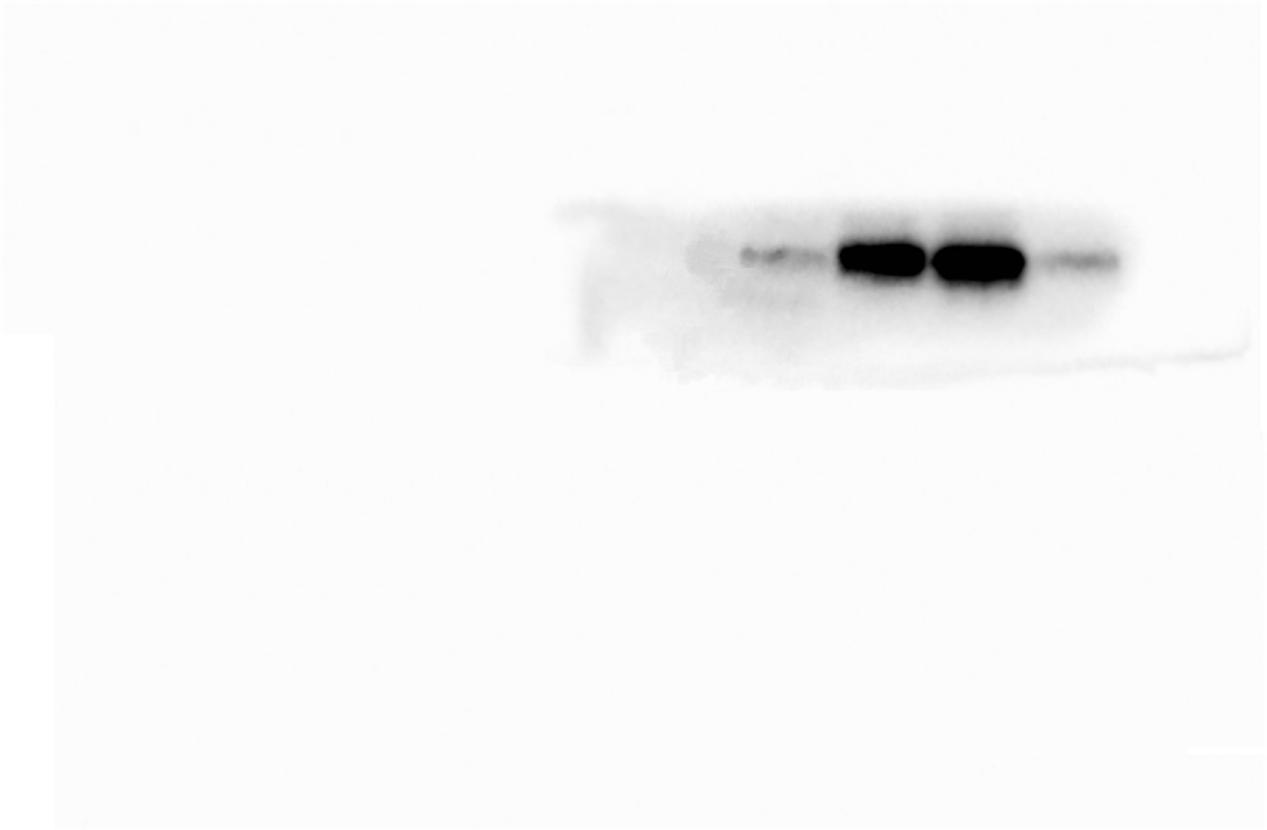


CD44 A


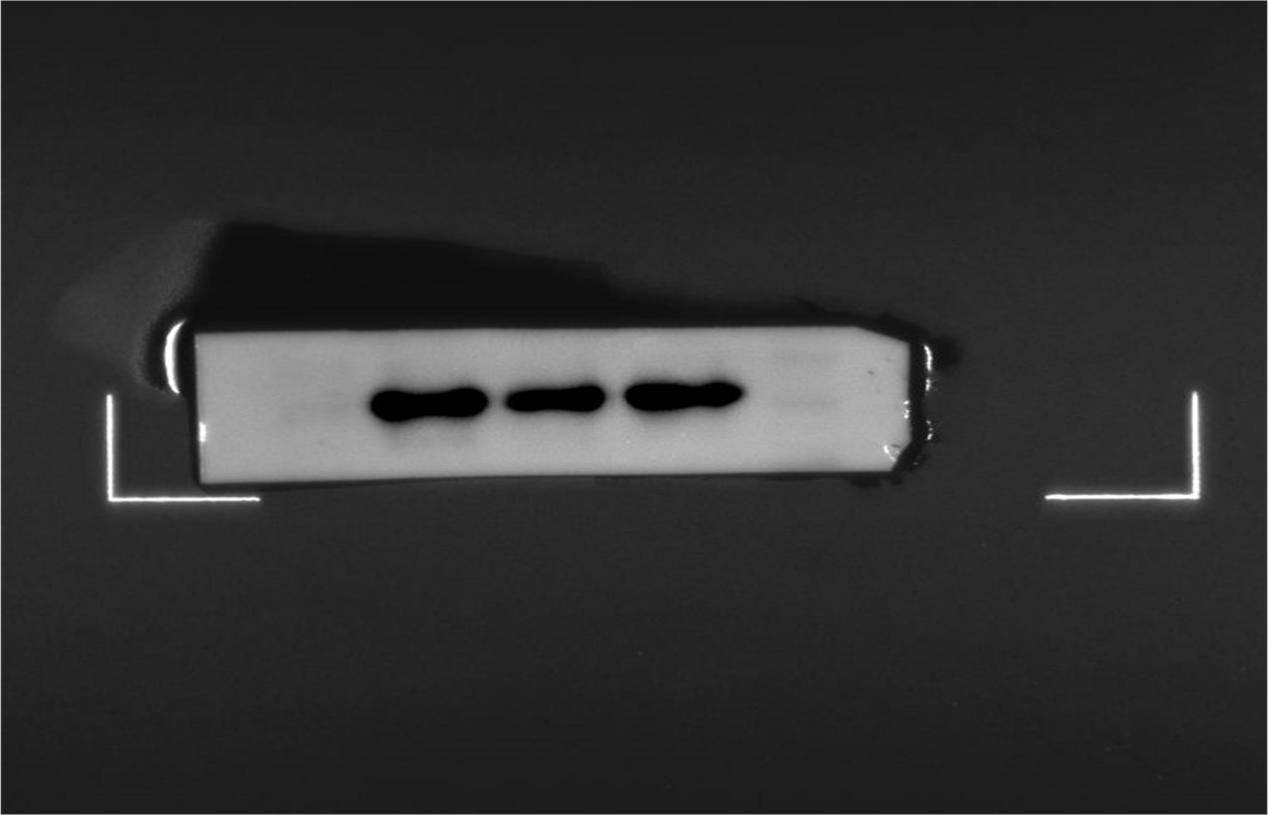


**GAPDH A**


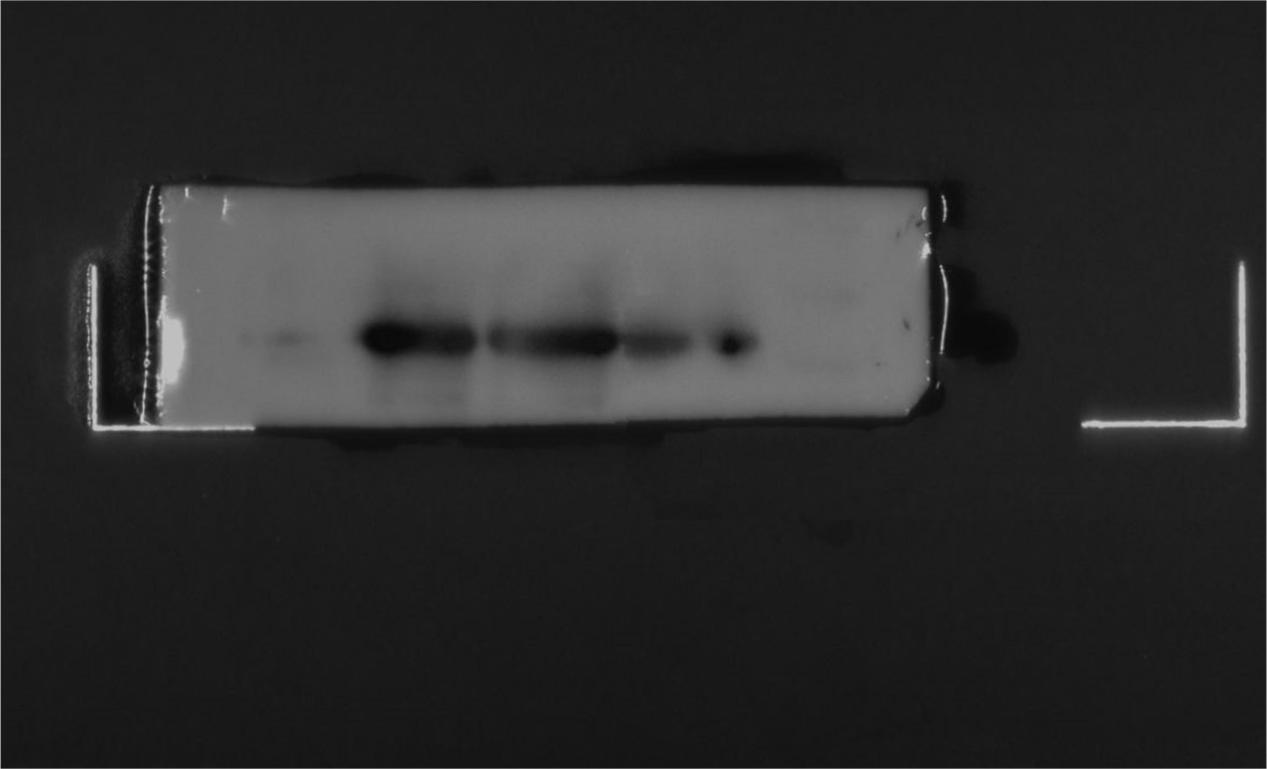


CD44 B

**
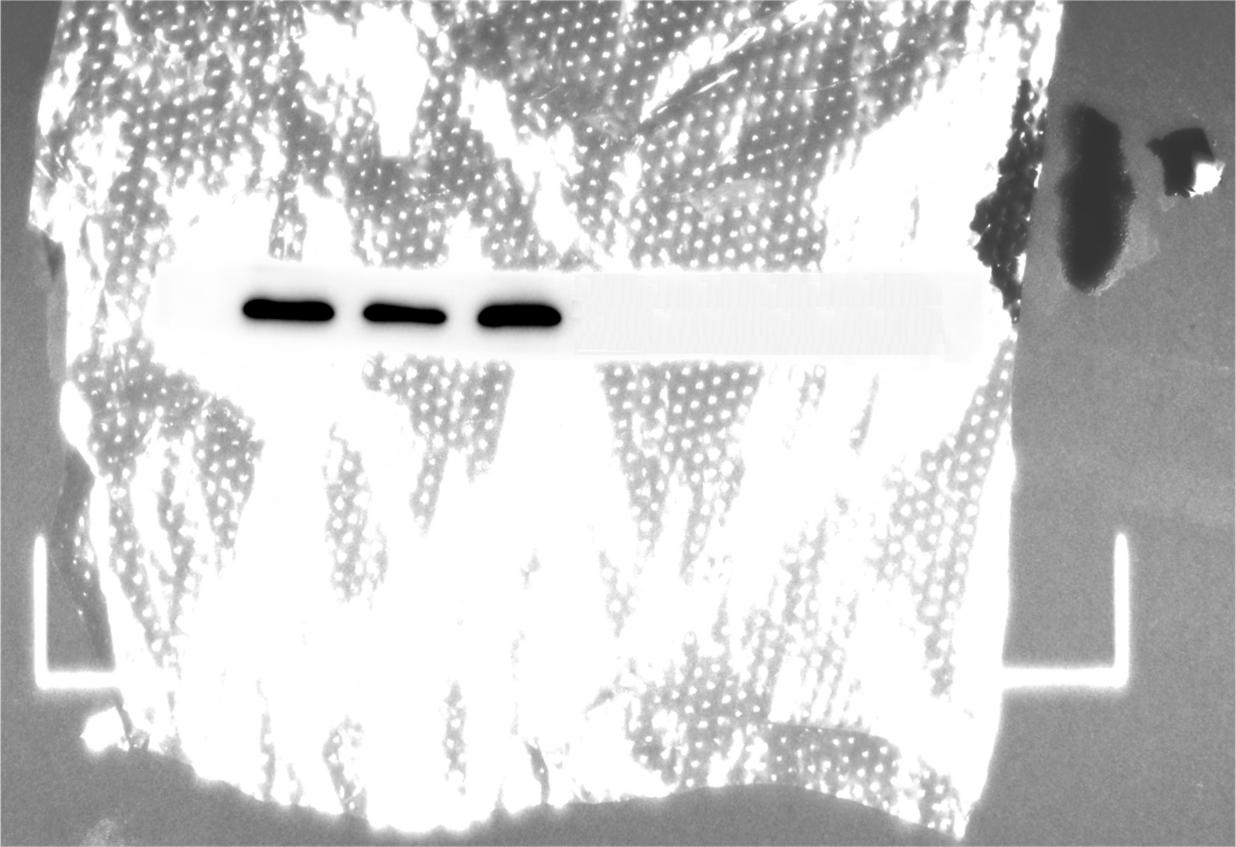
**

**GAPDH B**

Supplement: Supplementary file 4 — Additional file 4: Figure 4. The miR-138-5p negatively regulates CD44 protein expression. [file 12885_2023_10738_MOESM4_ESM.docx]

**Figure.7.** CD44st gene sequencing


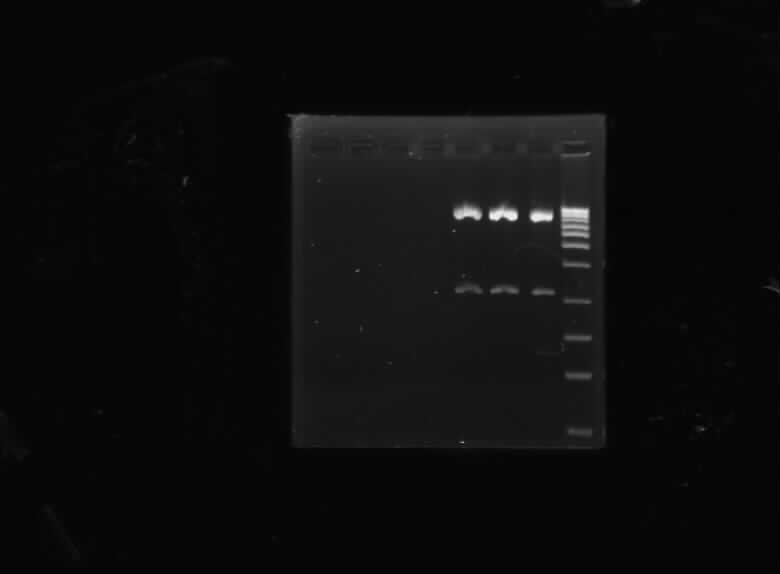

Supplement: Supplementary file 6 — Additional file 6: Figure 7. CD44st gene sequencing. [file 12885_2023_10738_MOESM6_ESM.docx]
