# Supplementary material for: Negative regulation of CD44st by miR-138-5p affects the invasive ability of breast cancer cells and patient prognosis after breast cancer surgery
Source: BMC Cancer. 2023 Mar 24;23:269. doi: 10.1186/s12885-023-10738-0 (PMC10037889; doi:10.1186/s12885-023-10738-0)
Supplement: Supplementary file 5 — Additional file 5: Figure 6. The miR-138-5p and CD44st mRNA expression in breast cancer and paracancerous tissues. [file 12885_2023_10738_MOESM5_ESM.docx]

**Figure.6.** The miR-138-5p and CD44st mRNA expression in breast cancer and paracancerous tissues.

Semi-quantitative PCR agarose electrophoresis plots of different tissues. (1–5：Breast cancer tissues and paracancerous tissues from five different patients , M: Marker)


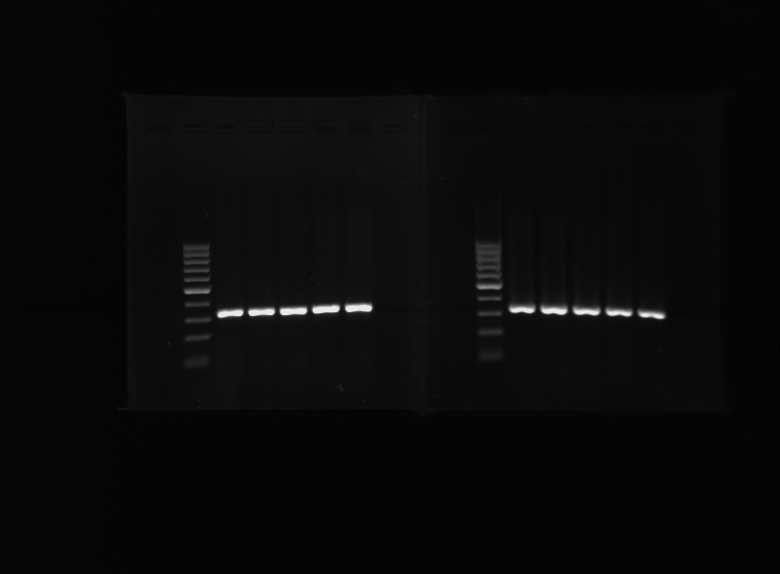


Bactin-cancer tissue and para-carcinoma


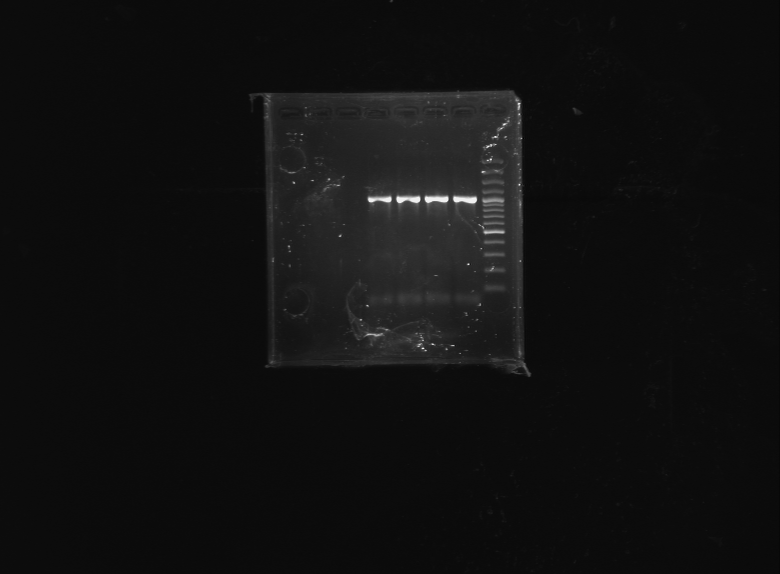


cancer tissue CD44st


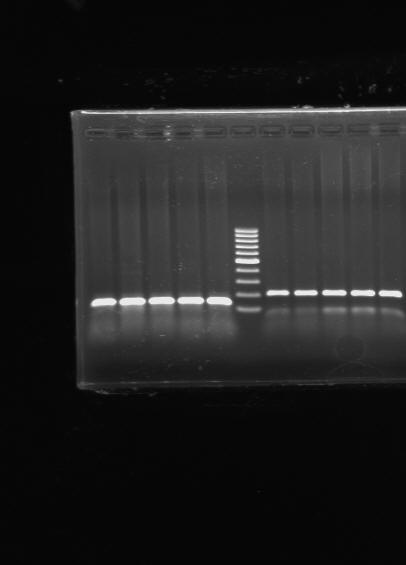


Mir-138-5p cancer tissue and para-carcinoma tissue


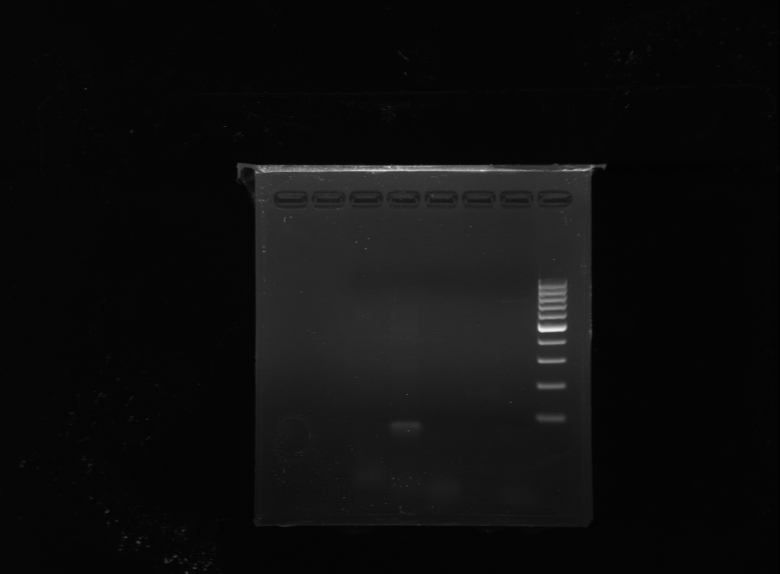


para-carcinomaCD44st
